# Book Reviews

**Published:** 1800-02

**Authors:** 


### CRITICAL RETROSPECT OF MEDICAL AND PHYSICAL LITERATURE.

[FOREIGN AND DOMESTIC.]

### MEDICAL PHILOSOPHY.

*Phyſiologie philoſophiſch bearbeitet:* i. e. A Philoſophic Syſtem of Phyſiology, by CHARLES CHRISTIAN ERHARD SCHMID, Profeſſor of Divinity at Jena. Volume Firſt, 1798. xxxiv. and 362 pp. Volume Second, 1799. viii. and 670 pp. 8vo. (price two rixdoll. and 18 groſch, or 11s. in ſheets) *Jena*, in the ſhop of the Univerſity.

THE Author of this elaborate work, though engaged in the active purſuits of a different profeſſion, has here ventured upon an inquiry, not only the moſt important, but likewiſe the moſt difficult that could have been undertaken in the whole department of medicine; he is therefore juſtly entitled to the approbation of the medical world, even though he ſhould not have ſucceeded in every part of this arduous attempt.

Prof. Schmid. has, on former occaſions, diſtinguiſhed himſelf among the German literati by his excellent philoſophical treatiſes on a variety of ſubjects, but eſpecially by his commentaries on the Critical Syſtem of Philoſophy, of which he is an able and ſtrenuous advocate. It would, however, be foreign to explain, in this place, the nature and tendency of his philoſophical writings, from which thoſe perſons, who are apt to confound the Critical Syſtem with others of a political nature, might eaſily learn how far *medicine* has been the object of theſe inquiries, and whether it is likely to derive from them any practical advantages. Yet, in order to characteriſe the ſpirit of theſe volumes, we ſhall only obſerve, that the reverend author writes in the moſt unaſſuming language; that he treats others who differ from him in opinion with the greateſt candour and liberality; that he does juſtice to the merits of his predeceſſors, even on thoſe occaſions where he is obliged to refute their errors; and that he never deviates from the opinions of other phyſiologiſts, without ſupporting his propoſitions by arguments, at once evident and concluſive.

If we conſider medicine as a ſcience, or as a ſyſtem of rules, it unqueſtionably, with all its collateral and auxiliary branches, forms a principal department of phyſics, or experimental philoſophy. The knowledge of medicine, at preſent, however, conſiſts chiefly and almoſt entirely, of a collection of individual obſervations ſyſ-

tematically arranged; from which useful rules, confirmed by experience, have been deduced for practical purposes. Indeed, it must be allowed, that we are not yet in the possession of scientific proofs or analytical demonstrations of these rules, so that we might reduce them to first and general principles: our induction for ascertaining their reality and occasional application cannot be derived from arguments sufficiently established in theory; and consequently such rules, hitherto, have had no *scientific* but merely a *technical* value and character.

Medicine, on the other hand, considered as an art, is still in its infancy; an assertion which no candid or honest practitioner will attempt to controvert. Even for the most valuable therapeutical, and especially the dietetic, discoveries and improvements, we are more indebted to accidental observations and analogical conjectures, than to a well-established or scientific theory. The *modus operandi*, of all the remedies employed in therapeutics, as well as the articles of diet, is so far inexplicable and obscure, that the whole difference between those which are called *rational*, and others which, by way of pre-eminence, are termed *specific*, depends merely on the compass of a rule, by which the technical application of the remedy is in every instance determined. So long, however, as medicine has, and is obliged to have, recourse to specific remedies, it must necessarily remain an empirical art. And in spite of those persons who, from a mistaken zeal for the honour of their profession, are anxious to vindicate its imperfections, it still remains destitute of scientific principles, and is in quest of a systematic form.

Notwithstanding the defects of what has hitherto, by usurpation, been called medical science, we may discover a cogent and constant desire in the human mind to reduce the phænomena of organized animal bodies to general principles; to explain from these, by scientific deduction, the most suitable technical method, not merely in an empirical but philosophic manner; to vindicate our medical treatment *à priori*, from the general laws of nature; and thus to effect a gradual, though indissoluble, connection between a scientific theory and practice. Every attempt hitherto made to establish a medical theory, as well as the present, is directed to that ultimate object. What Prof. Schmid terms "*Physiology treated philosophically*," is, in the work itself, called *Zoonomia*. Physiology ought to be the true basis of all medicine; consequently, it should comprise the Natural Philosophy of the human body; a system of laws relative to its corporeal powers; a more immediate application of the general doctrines of *organic* and *animal* nature to the particular organization of man, and to the peculiar relation which his structure, as well as his animal economy, bears to the human mind; this, however, can be attempted in so far only as we possess a knowledge of the mind and its operations. Physiologists generally define this science to be the doctrine comprising the destination and functions of the parts of the human body, or the knowledge of its healthy state; this definition is not only extremely wavering and undetermined, but it is obviously defective and inaccurate. They in this manner confound zoonomy with a very limited and imperfect zoology; and the real execution of their physiological works does not even correspond to this circumscribed plan. Let us, for instance, make the experiment, and separate from the best elementary books on physiology we possess, whatever relates to mere zoography, and we shall find that the stock of true physiological knowledge, such as is the real and peculiar property of that science, is very confined and imperfect. And this small remainder chiefly consists of zoology, is limited merely to the healthy state of the body, and takes no notice whatever of the relations subsisting between other objects of nature and the changes to which animals are subject; though it must be admitted, that it is impossible to acquire a philosophical, or even an accurate and connected historical knowledge of those changes, without having studied the relations between animate and inanimate nature.

In a strict sense of the word, therefore, even the best works we possess on physiology, furnish us with little physiological knowledge. They scarcely contain distinct ideas of organization and animal œconomy, of life and vital powers, properly unfolded; in short, physiologists have uniformly neglected to treat of the laws by which the operations of animality are regulated; of their relations among themselves, and the influence which Nature produces upon them in consequence of her internal and external agency; of the principles of pathology, derived from organized animal nature; of the preservation of health and prolongatiou of life; of therapeutics, materia medica, &c.

In the present work, the author endeavours to ascertain the true and accurate limits of physiological science, and to give it that systematic form of which it is susceptible. He has carefully compared and made use of all the facts and observations collected by his predecessors; he has exhibited general ideas and principles abstracted from a number of scattered remarks and experimental methods of cure, in order to combine them into a simple theory; in short, he has boldly and successfully attacked every species of medical dogmatism, while he has unfolded and solved many paradoxical and contradictory assertions of theorists: by such attempts only, we may hope to obtain, at length, a complete system of physiology.

In the Introduction, Prof. Schmid gives the definition of this science, its objects, limits, and form; its origin, history, and methodical study. I. *A Philosophic View of Physiology*, or, strictly speaking, *Zoonomy*, the Science of the Laws of Animal Nature. The Zoonomia of Dr. Darwin, with all its undeniable merit, does not perfectly correspond to the idea which this term implies according to its etymology, and the analogy it bears to words of similar construction: yet, we are not at this moment in possession of any work, whether foreign or domestic, which is better calculated to explain the doctrine of vital power. But the work of Proffessor Schmid was, nevertheless, the first which gave a distinct idea of this science, while it conveyed an appropriate term term to diftinguifh it from others. Perhaps, a future inquirer, endued with equal talents, will be placed in a more favourable fituation to form an highly interefting and fcientifically arranged fyftem, from the fcattered writings of Natural Philofophers, Chemifts, Anatomifts, Zoologifts, Phyfiologifts, and Medical Practitioners.—II. *Definition of Zoonomy.* It is the fcience of animal nature, or of the nature of living beings: it defcribes its object in a general manner, while it marks the mode and form of the knowledge we acquire of fubjects arranged under certain laws.—III. *Object of Zoonomy.* This cannot be any other but life, confequently animal nature, which originally and perceptibly is difcoverable only within ourfelves, fince it refts upon an immediate internal perception. Hence, the attention of the zoonomift is principally directed to the moft dignified and improved animal nature in man, which befides difplays, in the greateft order and harmony, the whole amount of animated organization and power: it is, on this account, that the organic ftructure of man affords the moft proper fcale for inftituting comparative reflections on all other fpecies of animals.

[To be continued in our next Number.]

*A New Syftem of Phyfiology, comprehending the Laws by which animated beings in general, and the Human Species in particular, are governed in their feveral ftates of health and difeafe; by* RICHARD SAUMAREZ, Surgeon to the Magdalen Hofpital. The Second Edition. Two Volumes Octavo, pp. 890. London. Cox, Johnfon, &c.

In the preface, the author informs us that "the efpecial object of "the enfuing work is to explore the nature of the principle of life, "and to affert its power; to inveftigate the attributes of organized "life, as the inftruments by means of which the phænomena of "organic action are accomplifhed, and the final caufe of animated "exiftence is attained."

Some of the fentiments, peculiar to this ingenious and indefatigable author, have already been laid before our readers in Volume II. pp. 242 and 321, &c.—Although we cannot coincide with him in all his opinions, we are convinced that no phyfiologift will read the work, without deriving from it pleafure and improvement.

*Three Lectures upon Animal Life, delivered in the Univerfity of Pennfylvania; by* BENJAMIN RUSH, M. D. Profeffor of the Inftitutes of Medicine, and of Clinical Practice in the faid Univerfity. Publifhed at the requeft of his Pupils, 8vo. pp. 84. *Philadelphia.* Dobfon.

From a fhort but manly preface, the learned medical veteran of America obferves, that although he did not think thefe Lectures fit for the public eye, he has, in confequence of the repeated application of his pupils, complied with their requeft, and been prevailed upon to commit them to the prefs, in their prefent imperfect ftate.

ftate. He farther, with a laudable modefty, difclaims the merit "of being the author of the great and original conception upon "which they are founded. I have done," fays he, "but little "more than carry the hod to affift in completing part of a fabric, "the foundations of which were laid by two of the moft diftin-"guifhed mafter builders in medicine in the eighteenth century."

In a fpirited and well-written introduction, the author unfolds the principle and extent of his lectures, in the following words:

"Some of thefe fubjects will be new in lectures upon the inftitutes of medicine, particularly thofe which relate to morals, metaphyfics, and theology. However thorny thefe queftions may appear, we muft approach and handle them; for they are intimately connected with the hiftory of the faculties and operations of the human mind; and thefe form an effential part of the animal œconomy. Perhaps it is becaufe phyficians have hitherto been reftrained from inveftigating and deciding upon thefe fubjects, by an erroneous belief that they belong exclufively to another profeffion, that phyfiology has fo long been an obfcure and conjectural fcience.

"In beholding the human body, the firft thing that ftrikes us is its LIFE. This, of courfe, fhould be the firft object of our inquiries. It is a moft important fubject; for the end of all the ftudies of a phyfician is to preferve life; and this cannot be perfectly done, until we know in what it confifts.

"I include in animal life, as applied to the human body, *motion*, *fenfation* and *thought*. Thefe three, when united, compofe perfect life. It may exift without thought or fenfation; but neither fenfation nor thought can exift without motion. The loweft grade of life probably exifts in the abfence of even motion, as I fhall mention hereafter. I have preferred the term *motion* to thofe of ofcillation aud vibration, which have been employed by Dr. Hartley in explaining the laws of animal matter; becaufe I conceive it to be more fimple, and better adapted to common apprehenfion.

"In treating upon this fubject, I fhall firft confider animal life as it appears in the waking and fleeping ftates in a healthy adult, and fhall afterwards inquire into the modification of its caufes, in the fœtal infant, youthful and middle ftates of life, in certain difeafes, in different ftates of fociety, in different climates, and in different animals."

After this introductory analyfis, the author lays down three general propofitions, from which we extract the following particulars, and occafional illuftrations:

"I. Every part of the human body, the nails and hair excepted, is endowed with fenfibility, or excitability, or with both.

"II. The whole human body is fo formed and connected, that impreffions made, in the healthy ftate, upon one part, excite motion, or fenfation, or both, in every other part of the body.

"III. Life is the EFFECT of certain ftimuli acting upon the fenfibility and excitability, which are extended in different degrees of every external and internal part of the body. Thefe ftimuli are as neceffary to its exiftence as air is to flame. Animal life is truly, to ufe the words of Dr. Brown, "a forced ftate." I have faid, the *words* of Dr. Brown; for the opinion was delivered by Dr. Cullen, in the Univerfity of Edinburgh, in the year 1766, and was detailed by me in this fchool, many years before the name of Dr. Brown was known as a teacher of medicine. It is true, Dr. Cullen afterwards deferted it; but it is equally true, I never did; and the belief of it has been the foundation of many of the principles and modes of practice in medicine, which I have fince adopted. In a lecture which I delivered in the year 1771, I find the following words, which are taken from a manufcript copy of lectures given by Dr. Cullen, in the Inftitutes of Medicine: "The human body is not an automaton, or felf-moving machine; but is kept alive and in motion by the conftant action of ftimuli upon it." In thus afcribing *the difcovery of the caufe of life*, which I fhall endeavour to eftablifh, to Dr. Cullen, let it not be fuppofed I mean to detract from the genius and merits of Dr. Brown. To his *intrepidity* in reviving and propagating it, as well as for the many other truths contained in his fyftem of medicine, pofterity, I have no doubt, will do him ample juftice, after the errors that are blended with them, have been corrected by their unfuccefsful application to the cure of difeafes."

In this candid and honeft declaration, we at once difcover the genuine fpirit of the tranfatlantic philofopher, while we are taught a practical leffon, how to appreciate the merits of others, without becoming the inftruments of a party, or beftowing unlimitted praife upon a difcovery, merely becaufe it bears the femblance of novelty. The ingenious author next proceeds to remark, "that the action of the brain, the diaftole and fyftole of the heart, the pulfation of the arteries, the contraction of the mufcles, the periftaltic motion of the bowels, the abforbing power of the lymphatics, fecretion, excretion, hearing, feeing, fmelling, tafte, and the fenfe of touch, nay more, thought itfelf, are all the effects of ftimuli acting upon the organs of fenfe and motion. Thefe ftimuli have been divided into external and internal. The external are, light, found, odours, air, heat, exercife, and the pleafures of the fenfes. The internal ftimuli are food, drinks, chyle, the blood, a certain tenfion of the glands which contain fecreted liquors, and the exercifes of the faculties of the mind; each of which I fhall treat in the order in which they have been mentioned."—p. 8.

As a fpecimen of the original and entertaining manner in which the author treats of the different external ftimuli, we extract the following:

"*Sound* has an extenfive influence upon human life. Its numerous artificial and natural fources need not be mentioned. I fhall only take notice that the currents of winds, the paffage of infects through the air, and even the growth of vegetables, are all attended with an emiffion of found; and although they become imperceptible from habit, yet there is reafon to believe, they all act upon the body, through the medium of the ears. The exiftence of thefe founds is eftablifhed by the reports of perfons who have afcended cended two or three miles from the earth in a balloon. They tell us, that the ſilence which prevails in thoſe regions of the air is ſo new and complete, as to produce an awful ſolemnity in their minds. It is not neceſſary that theſe ſounds ſhould excite ſenſation, or perception, in order to their exerting a degree of ſtimulus upon the body. There are a hundred impreſſions daily made upon it, which, from habit, are not followed by ſenſation. The ſtimulus of aliment upon the ſtomach, and of blood upon the heart and arteries, probably ceaſe to be felt, only from the influence of habit. The exerciſe of walking, which was originally the reſult of a deliberate act of the will, is performed from habit without the leaſt degree of conſciouſneſs. It is unfortunate for this, and many other parts of phyſiology, that we forget what paſſed in our minds the firſt two or three years of our lives. Could we recollect the manner in which we acquired our firſt ideas, and the progreſs of our knowledge, with the evolution of our ſenſes and faculties, it would relieve us from many difficulties and controverſies upon this ſubject. Perhaps this forgetfulneſs by children, of the origin and progreſs of their knowledge, might be remedied by our attending more cloſely to the firſt effects of impreſſions, ſenſation and perception upon them, as diſcovered by their little actions; all of which probably have a meaning as determined as any of the actions of men or women.

"The influence of ſounds of a certain kind in producing excitement, and thereby increaſing life, cannot be denied. Fear produces debility, which is a tendency to death. Sound obviates this debility, and thus reſtores the ſyſtem to the natural and healthy grade of life. The ſchool-boy and the clown invigorate their feeble and trembling limbs, by whiſtling or ſinging as they paſs by a country church-yard; and the ſoldier feels his departing life recalled in the onſet of a battle by the noiſe of the fife, and of the poet's "ſpirit-ſtirring drum." Intoxication is frequently attended with a higher degree of life than is natural. Now, ſound we know will produce this with a very moderate portion of fermented liquor; hence, we find men are more eaſily and highly excited by it at public entertainments, where there is muſic, loud talking, and hallooing, than in private companies, where there is no auxiliary ſtimulus added to that of the wine. I wiſh theſe effects of ſound upon animal life to be remembered, for I ſhall mention it hereafter, as a remedy for the weak ſtate of life in many diſeaſes, and ſhall relate an inſtance, in which a ſcream ſuddenly extorted by grief, proved the means of reſuſcitating a perſon, who was ſuppoſed to be dead, and who had exhibited the uſual recent marks of the extinction of life. I ſhall conclude this head by remarking, that perſons who are deſtitute of hearing and ſeeing, poſſeſs life in a more languid ſtate than other people; and hence ariſe the dulneſs and want of ſpirits which they diſcover in their intercourſe with the world."

[ To be contінded in our next Number. ]

 CHEMISTRY.

